# Innovative Technologies to Improve Occupational Safety in Mining and Construction Industries—Part II

**DOI:** 10.3390/s25185717

**Published:** 2025-09-13

**Authors:** Paweł Bęś, Paweł Strzałkowski, Justyna Górniak-Zimroz, Mariusz Szóstak, Mateusz Janiszewski

**Affiliations:** 1 Department of Mining, Faculty of Geoengineering, Mining and Geology, Wrocław University of Science and Technology, 50-370 Wroclaw, Poland; pawel.strzalkowski@pwr.edu.pl; 2 Department of Geodesy and Geoinformatics, Faculty of Geoengineering, Mining and Geology, Wrocław University of Science and Technology, 50-370 Wroclaw, Poland; justyna.gorniak-zimroz@pwr.edu.pl; 3 Department of General Construction, Faculty of Civil Engineering, Wrocław University of Science and Technology, 50-370 Wroclaw, Poland; mariusz.szostak@pwr.edu.pl; 4 Department of Civil Engineering, School of Engineering, Aalto University, P.O. Box 12100, FI-00076 Espoo, Finland; mateusz.janiszewski@aalto.fi

**Keywords:** innovative technologies, occupational safety, mining, construction

## Abstract

Innovative technologies have been supporting the improvement of comfort and safety at work in construction and mining, which are classified as high-risk sectors, for many years. A two-part study (Part I and Part II) was conducted in which the implementation of innovative technologies was analysed and evaluated (opportunities and limitations). In Part II, the technologies employed in the work environment by employees to enhance their comfort and safety at work were analysed. These technologies encompass virtual and augmented reality, innovative personal and collective protective equipment, and exoskeletons. Following a thorough analysis of the extant scientific literature from the Scopus database, it was determined that there were research gaps that required attention. In addition to the evident advantages of enhancing the safety of workers, innovative technological solutions also engender numerous economic benefits for employers, which impact sustainable development in enterprises. In order to fully exploit the potential of modern technologies, it is necessary to continue their integration and overcome implementation barriers, such as the need for changes in education and training, adequate funding, and the development of safety awareness and culture in companies.

## 1. Introduction

Occupational safety and health (OSH) in mining and construction is of particular significance in these industries, which are characterised by a high risk of accidents and injuries. With the progression of technology, novel prospects emerge for the automation of technological processes and the enhancement of employee safety. The introduction of modern technologies is imperative for enhancing working conditions and cultivating a proactive safety culture.

In our two-part series, innovative technologies for improving occupational safety in mining and construction are understood as new or significantly improved technologies, tools, systems, or processes that introduce novelty, bring economic or social benefits and are effectively implemented in practice, leading to advances and breakthroughs in various aspects of occupational safety and health.

The integration of modern technologies has been demonstrated to have a direct and positive impact on employee safety. Additionally, they enhance workers’ awareness and commitment to OSH regulations, thereby fortifying their capacity to manage crises, hazardous incidents, and accidents through the provision of timely information, realistic training, and effective support tools. As demonstrated by Al-Bayati [1] and Sidani et al. [2], the integration of technology with direct human use has been shown to significantly raise employee awareness of OSH principles and contribute to the development of a safety culture in the workplace. The identification and elimination of hazards in the workplace have become fundamental tasks that require innovative solutions [3]. The introduction of appropriate tools and systems has been shown to reduce the risk of accidents and strengthen preventive strategies [4], which in turn promotes the development of an appropriate OSH culture in the workplace.

OSH culture, defined as a set of values, beliefs, and norms relating to safety, plays a key role in effective safety management in organisations [5]. In the process of cultivating a safety culture within an organisational context, it is imperative that employees are instilled with a sense of personal responsibility for the safety of the workplace. In the context of mining and construction, where work processes are often complex and demanding, the establishment of a positive safety culture becomes of even greater importance. The provision of support for educational and training initiatives for employees by team leaders has been demonstrated to engender heightened commitment to OSH regulations, which can ultimately result in a reduction in accidents [6]. The efficacy of safety training in enhancing awareness, encompassing the knowledge and skills of employees regarding OSH regulations, is a pivotal aspect [7].

It is evident that the mining and construction sectors are becoming increasingly resilient to accidents and dangerous incidents. This is primarily due to dynamic technological progress and growing awareness of the importance of shaping a safety culture. However, it is important to emphasise that the implementation of these technologies must be done in a considered manner and adapted to the particularities of the workplace, as this will influence their effectiveness and acceptance among employees [8]. In the context of the specific working conditions prevalent in the mining and construction industries, there is a need for the ongoing research and implementation of effective technical solutions. This necessity arises from the imperative to guarantee efficacious measures that ensure the protection of workers in the workplace. Moreover, contemporary training methodologies are progressively assuming an indispensable role within safety management strategies. Consequently, it is incumbent upon companies to invest in the development and implementation of tools that enhance employees’ competence in the field of safety and engender a substantial increase in safety levels.

**Figure 1:**
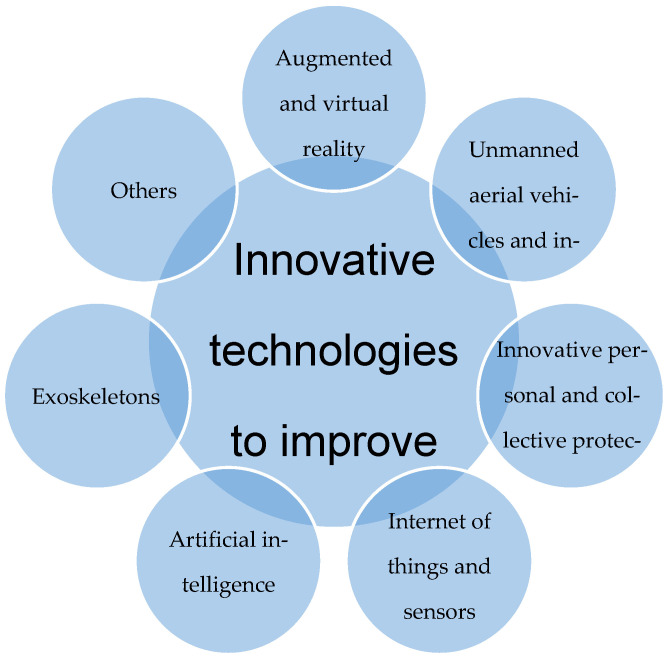
Innovative technologies to improve occupational safety in mining and construction industries (own study).

On the basis of the preceding discussion on the implementation of technology and its impact on safety culture, Figure 1 presents a framework that classifies technical solutions according to the manner in which they enhance work safety. The initial segment encompasses intelligent systems that are predicated on sensors and information technologies, including unmanned aerial vehicles, inspection robots, Internet of Things networks with smart sensors, and artificial intelligence platforms that primarily function to monitor working conditions, detect hazards in real time, and predict dangerous events. The primary objective of these platforms is to reduce the number of accidents and to safeguard the health of workers. The subsequent section delves into the technologies employed by workers in the workplace or during training. These include augmented and virtual reality, personal protective equipment for both individuals and groups, and exoskeletons. The objective of these technologies is to safeguard personnel from direct hazards, enhance work comfort, and educate personnel on safe behaviours through realistic, immersive instructions.

The aim of this article (Part II) is to attempt to systematise and analyse the most significant modern technologies for improving occupational safety that are used or can be implemented in the mining and construction sectors and are used directly by humans in the work environment or in the training process. To be more precise, Part I dealt with system technologies (drones and inspection robots, Internet of things and sensors, artificial intelligence), while Part II focuses on technologies used directly by employees (augmented and virtual reality, personal and collective protective equipment, exoskeletons). Solutions that are already functioning as stand-alone techniques are analysed, as well as the possibilities of combining them. The emphasis placed on the identification of technological development directions that have the potential to contribute to a reduction in the number of accidents at work, an increase in the effectiveness of occupational safety management, and an improvement in the quality of training and supervision of compliance with OSH procedures is particularly noteworthy. In Section 4, a comparative table is presented which details all modern technologies that have been selected for the study. This facilitated the discussion of the combination of the technologies discussed in this section of the article with all other technologies, including those discussed in Part I. These considerations have enabled the identification of practical examples of technological implementations, the identification of barriers to their implementation, and the formulation of recommendations for further research and development of technologies to improve working conditions and protect the life and health of workers in both sectors analysed.

Mining and construction are examined together in this study because both industries are characterised by high occupational risks, complex technological processes and demanding working environments that necessitate continuous improvements in safety management. While their specific working conditions differ, they share common challenges such as operating in confined or hazardous spaces, exposure to dynamic natural or structural factors and the need for advanced training and protective solutions. A joint analysis of these sectors not only reveals their differences but also uncovers transferable practices and synergies that can facilitate the adoption of innovative technologies across both fields. By adopting a broader comparative perspective, this publication highlights the interconnections and complementarities among different groups of technologies, providing a more comprehensive understanding of how they can collectively enhance occupational safety and operational efficiency in mining and construction.

## 2. Materials and Methods

**Figure 2:**
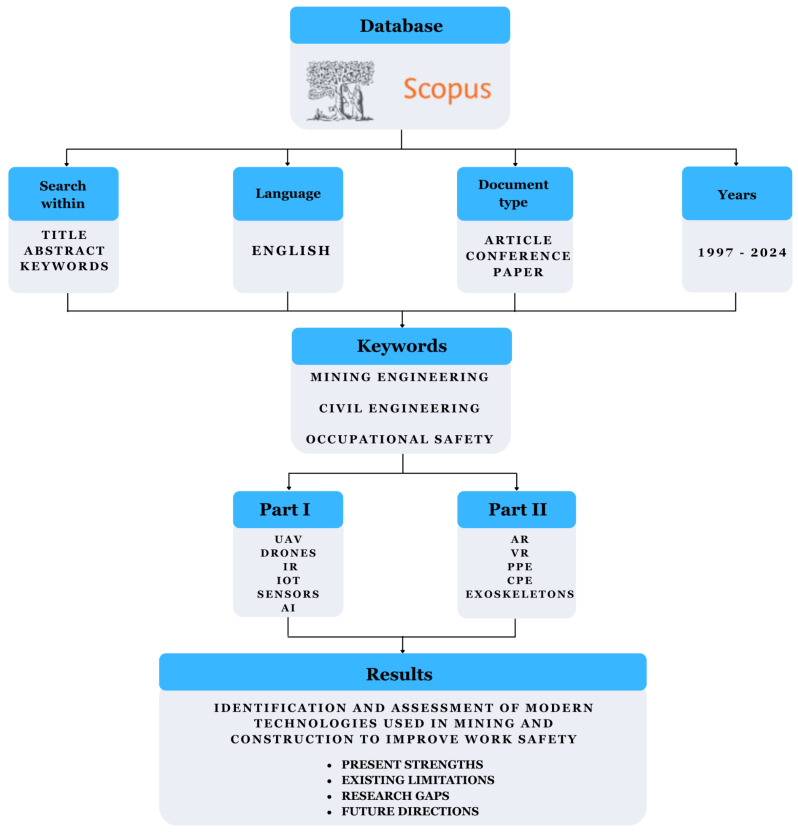
Diagram of the methodology of the conducted research (own study).

The literature review employed a desk research methodology, whereby research is conducted from behind a desk. Also known as secondary analysis, it involves collecting, analysing and interpreting published data. In the context of a literature review, desk research relies on foundational sources such as academic articles, reports, books, statistical data and publications from research institutions. This method enables the efficient and rapid acquisition of information, eliminating the need for field research [9,10]. A key element of desk research is critically analysing and evaluating the reliability, validity and relevance of sources to the research topic. Despite the fact that desk research allows specific research problems to be answered with little financial and organisational effort, it is still an underutilised analytical technique in research [11,12]. To illustrate the methodology undertaken and the subsequent steps of conducting research on the issue, the following diagram is presented (Figure 2). The same research methodology was used in both parts of the articles (Part I and Part II). The only difference in the methodology is the keywords used in the Scopus search.

**Figure 3:**
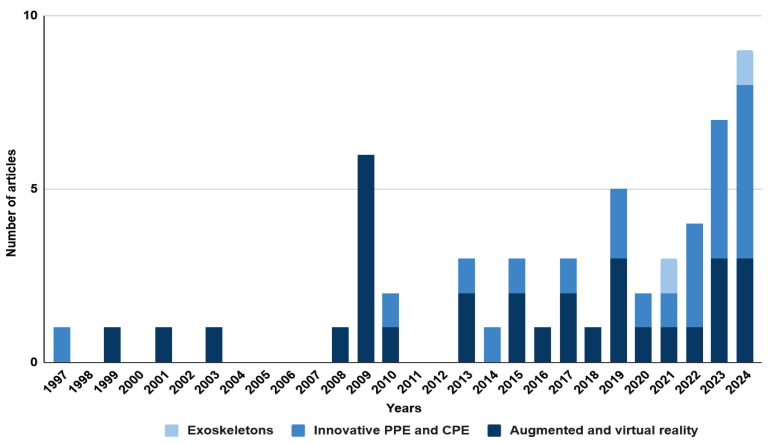
The number of literature results for a given technology in mining engineering in specific years (own study based on Scopus database).

**Figure 4:**
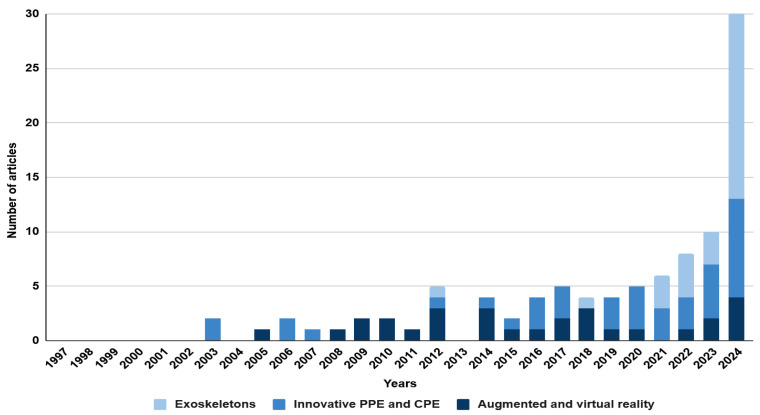
The number of literature results for a given technology in civil engineering in specific years (own study based on Scopus database).

Source analysis was carried out based on the keywords established in Table 1. Two sets of keywords were selected: ‘mining engineering’ and ‘civil engineering’. The research was conducted using the Scopus database. The literature review covered the period from 1997 to 2024 and included only original scientific articles and conference papers written in English. As a result, a total of 154 records were obtained. All the selected publications were analysed in detail, and those not related to occupational safety in mining and civil engineering were excluded. Based on these publications, the technologies used to improve occupational safety were characterised. The analysis revealed an increasing interest in the topic, which may suggest the development and popularity of innovative technologies and their application in various areas of mining and civil engineering. The above conclusion is illustrated in the graphs (Figure 3 and Figure 4). The only difference in the methodology is the keywords used in the Scopus search.

A review of the literature was conducted to assess the potential for combining technologies to improve occupational safety. Areas for combining technologies were proposed, as were limitations of inter-technological integration.

## 3. Results

### 3.1. Augmented and Virtual Reality

Virtual and augmented reality (VR and AR) are advanced technologies that create interactive, three-dimensional environments simulating the real world. In the context of high-risk sectors, such as mining and construction, the use of these devices has emerged as a pivotal strategy for enhancing occupational safety standards. The utilisation of VR and AR facilitates the presentation of objects and the recreation of realistic action scenarios, in addition to conducting training in conditions that approximate real-life scenarios, whilst eliminating the exposure of workers to actual hazards. The technology is utilised in the domains of hazard analysis, evacuation planning, the testing of emergency procedures, and the training of personnel in the operation of complex machinery and systems. The utilisation of virtual reality and augmented reality in training regimens has been demonstrated to facilitate the acquisition of experiential knowledge and the development of competencies in a controlled setting, thereby markedly diminishing the likelihood of accidents and operational errors. Furthermore, the utilisation of virtual simulations facilitates the identification of deficiencies in extant health and safety systems, thereby enabling their continual enhancement. The integration of virtual and augmented reality into investment design and monitoring processes has been demonstrated to engender enhanced safety measures and concurrently effectuate a reduction in costs associated with occupational accidents and periods of downtime. It is evident that VR and AR not only promote worker well-being, but also contribute to environmental protection by reducing incidents that could lead to contamination or damage to infrastructure [13,14,15].

It is imperative for graduates to possess a fundamental understanding of the equipment and machinery employed within the industry upon their entry. This is primarily due to the fact that they have received training on outdated equipment, due to the lack of modern equipment in educational institutions. The primary impediment pertains to constrained financial resources, which hinders the recruitment of qualified personnel, their ongoing professional development, and the acquisition of contemporary teaching materials and technological resources. Universities are unable to keep up with industry developments; therefore, the education sector must undergo a transformation. Technologies such as AR and VR are playing an increasingly important role in practical learning, enabling the creation of realistic simulations and the development of spatial skills and understanding of complex technical concepts [16,17].

Virtual reality can be defined as a system that, through the combination of devices and computer programmes, enables the user to have an immersive experience in the digital world, thereby stimulating their perception [18]. The utilisation of visualisation techniques has been demonstrated to facilitate the comprehension of complex concepts by rendering them graphically, thereby enabling their perusal at any time and in any location. The efficacy of VR and AR training has been demonstrated for a wide range of users, irrespective of age or experience, and has been met with a positive response. In order to realise the potential of VR and AR, it is necessary to utilise devices that facilitate the effective use of the software. The primary benefits of VR and AR are realism and immersion, which engender a sense of inhabiting a simulated or augmented reality as if it were reality itself. The development of graphics engines and software tools is facilitating the creation of interfaces in VR and AR environments. These interfaces utilise three-dimensional graphics to facilitate intuitive interaction with both virtual and real objects [19,20,21,22].

#### 3.1.1. Mining Engineering

In recent years, there has been an increasing use of VR and AR technologies in the mining industry. Visualisation has been demonstrated to facilitate the accurate consideration of past experiences and events, thereby enabling more informed decision-making. Furthermore, the utilisation of remote control technology has been demonstrated to facilitate the process of mapping virtual models to the actual mine layout [23,24,25]. The utilisation of virtual reality (VR) and augmented reality (AR) simulation has emerged as a highly effective training and educational tool, facilitating the emulation of perilous and unorthodox conditions within mining environments, and the resolution of intricate challenges. The potential for the successful application of VR and AR technologies to mining operations is significant. One such example is the employment of interactive simulators for the training of operators of multiple excavators. The findings suggest that immersive simulator training can yield quantifiable, short-term performance enhancements for experienced multi-excavator operators [26]. In the contemporary context, virtual reality (VR) and augmented reality (AR) are being employed with considerable efficacy in the mining industry for the purposes of data visualisation, accident reconstruction, simulation applications, risk analysis, hazard awareness and training [21,27,28,29].

In the contemporary mining industry, employees are expected to demonstrate high levels of professional competence and the capacity to effectively function in challenging environments. Innovative virtual and augmented reality technologies have emerged as pivotal tools in the training and upskilling of mining personnel. Realistic simulations of the work environment have been shown to facilitate effective training for both students and industry professionals. The integration of VR and AR is becoming increasingly central to digital transformation, with significant implications for the manufacturing and educational sectors. The university’s collaboration with industry fosters the development of curricula, specialised courses and interdisciplinary research, thereby promoting the establishment of a digital culture and preparing personnel to face future technological challenges [30].

Augmented and virtual reality are finding increasing use in mining, particularly in the area of workplace safety. The use of augmented and virtual reality has enabled the creation of realistic simulations of underground hazards, facilitating the training of workers in a controlled environment without the need for exposure to actual risks. The implementation of these technologies facilitates the dissemination of real-time data, including the location of emergency exits, hazardous areas and machine parameters, directly to the worker’s field of vision. This is achieved through the use of specialised glasses or mobile devices. Consequently, there is an enhancement in awareness of potential hazards and the capacity to respond expeditiously in emergency situations. Furthermore, AR and VR have been demonstrated to facilitate the identification of potential failures in inspection and maintenance processes, thereby contributing to the prevention of accidents. The mining industry is characterised by a high risk of accidents; consequently, the provision of effective safety training for employees is of paramount importance. Virtual and augmented reality offer an engaging and realistic environment in which to simulate hazardous situations, resulting in better recall and understanding of procedures [14,31,32,33,34].

The utilisation of VR and AR facilitates the safe exploration of risks within a controlled environment, thereby enabling employees to learn from errors in a virtual or augmented setting without the concern of tangible repercussions. The most significant advantage is the capacity to replicate situations that are challenging to simulate in reality. For instance, virtual and augmented reality facilitate the simulation of complex scenarios, such as an explosion in a mine, which would be impractical or prohibitively expensive to recreate in a physical setting. It appears that enhancing engagement and motivation is also of significance. The interactive nature of VR and AR has been demonstrated to enhance the appeal and engagement of training programmes. Furthermore, the utilisation of VR and AR in research and analysis has been shown to facilitate the examination of ergonomics, risk factors and the evaluation of novel solutions within controlled environments.

Modern technologies, including 3D scanning, computer simulation and virtual reality, have enabled significant advancements in the design of underground workings. These technologies have been demonstrated to increase process reliability, reduce costs and improve safety. Virtual and augmented reality facilitate the realistic visualisation and testing of designs, thereby supporting more accurate planning and design, as evidenced by the development of mining machinery. Indeed, VR has been shown to be an effective tool in ergonomic analysis and assembly simulation [14,15].

#### 3.1.2. Civil Engineering

The utilisation of virtual and augmented reality in the construction industry is a growing trend, encompassing both the design stage and the execution of projects. Initially, the primary applications of VR and AR were in the field of project visualisation, aiding clients and developers in comprehending architectural concepts with greater ease. Subsequently, these technologies began to be used for simulating construction processes and work planning, which enabled optimisation and streamlining of construction workflows. Presently, VR and AR also play a significant role in the education and training of construction workers, as well as in the testing and implementation of innovative technological solutions [15,35,36].

The advent of emergent technologies has had a profound impact on the realm of construction safety. In particular, the efficacy of safety performance is enhanced through the utilisation of effective technologies such as artificial intelligence, virtual and augmented reality, Building Information Modelling (BIM), and wearable devices [37]. The BIM methodology has been adopted extensively in the construction, engineering, and architecture sectors. Augmented reality, as a developing technology, has gained popularity in recent decades, particularly within the construction industry. The primary applications of this software include the visualisation of structures and the creation of virtual models. These technologies have been demonstrated to facilitate enhanced risk management in projects and to promote collaboration with clients and other stakeholders [38].

Construction is a high-risk industry in which numerous accidents are often caused by human error. Traditional safety training methods frequently prove to be inadequate, thus rendering virtual reality an increasingly popular educational tool. The utilisation of augmented reality technology in the construction industry offers a potential solution to this issue. By employing AR, workers can access virtual instructions, hazard warnings, and danger zone markings directly on the construction site, thereby helping to minimise accident risks. Furthermore, the utilisation of VR and AR in simulated work environments facilitates the delivery of realistic safety training, thereby enhancing the preparedness of workers to respond effectively to emergency situations. Furthermore, this technology facilitates the execution of inspection processes, enabling the expeditious identification of irregularities and potential structural hazards without compromising the continuity of construction operations. The utilisation of virtual reality and augmented reality facilitates the emulation of hazardous scenarios without the necessity of exposing workers to genuine risks, thereby enhancing the efficacy of training and participant engagement [14,39,40,41,42].

Virtual and augmented reality have become pervasive in facility management and engineering education. These technologies facilitate realistic analyses of structural conditions, as evidenced by their application in building maintenance and bridge inspection projects. Furthermore, they facilitate learning through the use of immersive simulation environments, which have been shown to enhance spatial awareness skills. The utilisation of VR and AR in training professionals, such as construction machinery operators, has been demonstrated to be efficacious. This is achieved by enabling practitioners to hone their skills in a controlled and realistic environment, utilising virtual models and augmented instructions [14,15].

VR and AR have been used to facilitate safety training in various contexts, including working at heights, operating machinery, and hazard identification on construction sites. Furthermore, virtual and augmented reality technologies have been demonstrated to be efficacious in the field of hazard science, including the assessment of noise exposure and fall risks. A significant factor in recognising their potential is the ability to test human responses to threats, for example, through behavioural analysis in simulated high-stress situations. The utilisation of virtual reality and augmented reality has been demonstrated to facilitate enhanced communication among designers, decision-makers, and end users, thereby contributing to the optimisation of the design, planning, and lifecycle management of buildings. These technologies facilitate realistic project visualisation and early error detection. The integration of BIM, VR and AR environments has been demonstrated to enhance the design of structures and installations, as well as enable energy simulations. The utilisation of these tools in construction planning facilitates interactive analysis of on-site activities and optimisation of layout. Furthermore, these technologies facilitate workforce training and project management through the utilisation of realistic situational simulations, thereby enhancing the efficacy of decision-making processes and ensuring the safety of projects.

### 3.2. Innovation Personal Protective Equipment and Collective Protective Equipment

A review of the existing literature indicates that the implementation of both personal protective equipment (PPE) and collective protective equipment (CPE) is instrumental in enhancing safety in the construction and mining industries. The efficacy of these measures is contingent upon factors such as appropriate selection, ergonomics, and the awareness of the workforce. Innovative technologies, including smart PPE equipped with IoT sensors and vision systems based on deep learning, are transforming the manner in which protective equipment is monitored and enforced. In addition, it is imperative to manage risks through hazard assessments, to monitor the work environment (e.g., dust and noise emissions), and to strengthen safety culture through employee training and education. The advancement of automation and robotics in the mining industry has also been identified as a contributing factor to the reduction in individuals’ exposure to hazardous working environments.

#### 3.2.1. Mining Engineering

Occupational safety in mining is characterised by specific and complex hazards, and is a constant subject in research and innovation. The analysis of the publications presented here reveals a multifaceted approach to the role of personal protective equipment and collective protective equipment in minimising risks and improving working conditions for miners around the world. The cornerstone of ensuring safety in mining operations is predicated on a comprehensive understanding of the potential hazards that pertain to the specific nature of the work. Research on the development of industrial hygiene programmes in new copper mines [43] emphasises the significance of systematic risk assessment and the implementation of appropriate control measures, both in terms of PPE and CPE. Concurrently, an analysis of the aetiology of accidents in nickel mines [44] provides pivotal information for identifying areas in need of reinforced prevention, including the more effective utilisation of PPE and emergency medical services (EMS).

In the context of specific hazards such as dust, research into the spread of respirable dust during tunnelling [45] is pivotal to the effective design of ventilation systems and the selection of appropriate respiratory PPE. Monitoring levels of potentially harmful substances such as mercury and arsenic in the vicinity of abandoned mines [46] serves to underscore the long-term environmental and health consequences of such activities, and the necessity for continuous monitoring and the use of appropriate personal protective equipment (PPE) when necessary.

Traditional approaches to safety are complemented by innovative technologies. The notion that “the best PPE is the brain” underscores the significance of safety culture and worker awareness, which are indispensable for the effective utilisation of any form of protection. The advent of smart PPE, equipped with sensors and communication systems [47,48], engenders novel opportunities to monitor working conditions, workers’ locations and their health in real time, thereby significantly augmenting safety and the potential for rapid response in emergency situations.

The application of deep learning for the purpose of PPE detection [49,50,51] has precipitated a paradigm shift in the manner by which safety compliance is monitored, thereby enabling automated checks for the utilisation of requisite equipment such as helmets by workers. The integration of body position estimation with PPE detection [52] represents a significant advancement, providing more detailed information on worker location and activity, which is crucial in rescue operations.

Concurrently, advancements in robotics and the Internet of Things (IoT) [53] have enabled machines to execute hazardous tasks, thereby mitigating human exposure to risk. The utilisation of alert-enabled health and work environment monitoring systems [54] facilitates the early identification of potential hazards, thereby enabling the dissemination of pertinent information to workers, thus facilitating a swift response and the avoidance of potential accidents.

Nevertheless, the effective use of PPE is not without its challenges. Research on the ergonomics and usability of headgear PPE [55] highlights the importance of comfort and fit, which have a direct impact on the willingness and correctness of their use. An analysis of the influence of user characteristics on the dimensional tolerances of PPE [56] indicates the necessity for individualised equipment fit.

#### 3.2.2. Civil Engineering

Occupational safety in the construction industry remains a key challenge, and the use of personal protective equipment (PPE) and collective protective equipment (CPE) is the cornerstone of effective prevention strategies. A thorough analysis of the publications presented illuminates a range of aspects pertaining to their implementation, effectiveness and challenges in the construction industry worldwide.

A significant number of studies have underscored the necessity of investigating the underlying causes of accidents, with falls from height being a prominent example of such incidents [57]. These incidents are particularly prevalent and severe in the context of construction sites. It is imperative to comprehend the underlying causes to facilitate the design and implementation of suitable CPE, such as guardrails, safety nets or scaffolding systems. This understanding is crucial for determining the necessity of PPE, including safety harnesses and belay ropes, in particular circumstances. The methodology for the development of energy absorbers for safety ropes [58] indicates a continuous effort to improve personal protective equipment (PPE) technology to minimise the effects of potential falls.

The evaluation of potential hazards at various stages of construction, as evidenced in the context of residential construction in Estonia [59], constitutes a fundamental step in the identification of the locations and the nature of the most requisite CPE and PPE. In contrast, studies of workers’ perceptions of safety, for example, in Turkey [60], demonstrate that awareness and acceptance of the use of PPE have a significant impact on their effectiveness.

In the context of emergent hazards, such as contact allergies associated with novel technologies [61], there is an emerging need to identify appropriate PPE, such as protective gloves and skin-protective clothing. Furthermore, exposure to chemicals during the process of painting metal structures [62] necessitates the use of specialised respiratory and skin protective equipment (PPE).

Many publications focus on the use of modern technology to improve adherence and effectiveness of PPE use. Smart monitoring platforms, such as SmartHat [63], integrate sensors and alarm systems into workwear, thus enabling real-time monitoring and warning of potential hazards. The development of machine learning-based systems for detecting the absence of PPE [64] is a promising direction in automating inspections and ensuring that workers are using the required equipment. The integration of BIM with safety issues [65] creates opportunities for virtual analysis of construction sites, identifying potential collisions and planning safe work zones, resulting in more efficient use of CPE.

However, the implementation and effective use of PPEs and CPEs face various barriers. A study in New Jersey [66] reveals that small construction companies may encounter issues with cost, lack of awareness or insufficient enforcement. Similarly, an analysis of the implementation of the COVID-19 safety regulations in Ghana [67] demonstrates that economic and organisational factors have the capacity to impede the utilisation of even basic protective equipment. The issue of tailoring PPE to individual workers’ needs, including consideration of gender differences [68], is increasingly being raised. The issue of mismatched PPE is of particular concern, as it has been demonstrated to engender a state of physical discomfort, in addition to restricting movement, which can in turn result in a diminution of the workforce’s inclination to utilise the equipment.

### 3.3. Exoskeletons

In high-risk sectors such as mining and construction, the implementation of exoskeletons has been shown to result in a substantial reduction in work-related musculoskeletal disorders (WRMSDs), enhancing both comfort and efficiency. This is a primary contributing factor to absenteeism and reduced productivity [69,70]. Exoskeletons, which provide physical support during work activities, represent a promising technological solution to the problems resulting from repetitive and physically demanding tasks performed in suboptimal positions.

A survey [71] revealed the availability of more than 130 models from over 20 countries, 66% of which were aimed at industry, with only 14% claiming support for construction (mining was not analysed). All exoskeletons are general-purpose, supporting the spine and limbs.

#### 3.3.1. Mining Engineering

Despite the growing number of scientific publications on exoskeletons, their use in mining remains a rarely covered topic. Nevertheless, the utilisation of exoskeleton technology within the domain of mining engineering has materialised as a transformative advancement intended to enhance worker safety, productivity, and overall well-being in arduous environments. The field of mining is characterised by a unique set of challenges, including the potential for musculoskeletal disorders arising from the exertion of force through heavy lifting and the adoption of awkward postures. Exoskeletons are designed to mitigate these risks by providing physical assistance, thus improving ergonomics and reducing the physical strain on workers.

As Zeng et al. [72] demonstrate, a comprehensive topological design framework for mining exoskeletons is imperative, with emphasis placed on ergonomic considerations and the complexity of mine environments. This focus on ergonomics is supported by the work of Ali et al. [73], who note that active exoskeletons are capable of adjusting to a variety of tasks, thereby enhancing versatility and offering increased protection against physical strain. A notable finding is that a systematic review by Looze et al. [74] indicates significant muscle activity reductions of approximately 80% with active exoskeletons, further reinforcing their potential benefits in labour-intensive industries such as mining. In contrast, Zeng et al. [72] propose a design scheme for exoskeletons for mining, with a focus on the challenges of their design and functionality. While this publication provides valuable information for designers, it lacks data on practical implementation and challenges associated with real-world working conditions.

Despite the paucity of research in this area, advances in technology and increased interest in mine safety could contribute to the development of exoskeletons tailored to the industry. It is imperative to concentrate on research and pilot deployments in order to evaluate their suitability within the mining environment. Exoskeletons have been shown to provide physical support to workers, thereby enhancing safety and increasing productivity. However, the implementation of such systems is not without significant challenges, including the adaptation to confined spaces, the provision of sufficient power, and the resistance to inclement conditions.

#### 3.3.2. Civil Engineering

Construction workers are exposed to chronic overloads resulting from working in forced positions, leading to musculoskeletal injuries. The most prevalent of these are shoulder injuries associated with prolonged work over the head [75]. In response to this challenge, a sophisticated exoskeleton system equipped with advanced motion monitoring capabilities was developed. Studies [76] have validated the efficacy of this innovation in reducing exertion and discomfort, including in the lower back region. An evaluation of the viability of exoskeletons in various construction tasks was conducted by [77]. Since 2021, the results of practical research have been appearing. Exoskeletons have been utilised by concrete workers, steel fixers, fitters and general construction workers, providing coverage for the lower and upper limbs and the spine. For instance, a passive lower limb exoskeleton employed for the purpose of concrete spraying has been demonstrated to reduce stress levels [78]. In contrast, the exoskeletons used by fixers have been shown to reduce work time by 50% and alleviate back pain, although they have also been associated with an increase in chest discomfort [79,80,81]. It was also observed that pipe fitters experienced a reduction in spinal strain. A series of studies were conducted in laboratory conditions (e.g., ref. [76]) and on construction sites (in situ), with improvements in work comfort being identified in the latter (see also ref. [82]). The utilisation of back exoskeletons has been demonstrated to result in a reduction in lower back muscle activity [83], with a concomitant ergonomic risk reduction of up to 31.7% [84,85]. As demonstrated in [86], a reduction in lumbar strain on the spine has been confirmed among fixers. Exoskeletons of the upper limbs have been shown to reduce the activity of the shoulder muscles by up to 58% [87] and the forearm muscles by 5–6% [88]. The reduction in tension in the hand was 36%. In the study conducted by Mänttäri et al. [89], the utilisation of the upper limb exoskeleton during work at height resulted in a reduction in deltoid muscle activity by 32–46%. A decline in muscle activity of up to 27% has been documented among drilling workers [90]. A study conducted by Du et al. [91] involved 41 participants, and the results indicated that 66% of individuals would be willing to utilise an exoskeleton, particularly for tasks requiring overhead movement. A number of studies have been conducted to examine the impact of exoskeletons on cognitive function. For instance, ref. [92] demonstrated that exoskeletons can facilitate attention but compromise the precision of threat identification. As indicated by other research [84,93], there is a possibility of an increased cognitive load of up to 33%, which may have a detrimental effect on memory and situational awareness. Afolabi et al. [94] posited that chronic mental strain has the capacity to diminish productivity and well-being. In addition, ref. [95] found that exoskeletons can upset the balance of the body.

## 4. Evaluation of Modern Technologies for Improving Occupational Safety

### 4.1. General Characteristics and Future Research Directions

#### 4.1.1. Augmented Reality and Virtual Reality

Virtual and augmented reality technology has been identified as a significant tool in the teaching process, with considerable potential to support training in occupational health and safety [7]. This technology enables the simulation of complex and hazardous situations within a real work environment, without endangering the employee. Research findings indicate that training facilitated by virtual reality and augmented reality is both effective and positively evaluated by employees, with the potential to contribute to enhancing safety in the mining and construction industries [13,14].

The availability of virtual and augmented reality is increasing, and a variety of AR and VR products and content are reaching a wide audience. Despite the fact that AR and VR have been subjects of research and applications for decades, the rapid development of the technology means that there are still many unresolved issues that require further analysis [25].

The medium of VR, with its capacity for profound immersion in simulations and visualisations, has the potential to effect a revolutionary transformation in the work environment. It is evident that the advent of virtual reality has engendered a paradigm shift in the manner in which users interact with virtual environments. The technological sophistication of VR enables users to “move” into the virtual world, thereby facilitating the analysis of processes from any perspective. This technological advancement enables users to replay processes and move in time, thereby offering a more dynamic and immersive experience. Moreover, the virtual presence of colleagues obviates the necessity for physical meetings, thereby creating new opportunities for international cooperation. In the future, the nature of work will be transformed by the advent of VR technology, which will render physical location a moot point. The advent of sophisticated tools has enabled engineers and designers to engage in remote collaboration on three-dimensional models. Nevertheless, the integration of virtual reality within this process is accompanied by certain challenges. Notwithstanding this fact, engineers and scientists are optimistic about the potential of VR in the context of transforming the traditional workplace [96].

Virtual reality has been demonstrated to have great potential, but its implementation is not yet mature. The utilisation of VR technology has garnered significant recognition within the mining and construction industries. Its utilisation is pervasive, encompassing every phase of the business lifecycle, including design, planning, management, and the establishment of safe working environments. It is conceivable that in the near future, it will become a pivotal instrument not only for enhancing the efficiency of technological processes, reducing costs and maximising profits in the world’s most profitable enterprises, but also for facilitating the realisation of human potential.

Given the continuous technological and scientific progress that has been observed in recent years, it is reasonable to hypothesise that VR will become a key element in humanity’s expansion beyond our planet. The utilisation of augmented reality (AR) in domains such as mining and construction is gaining prominence, particularly with regard to enhancing occupational safety. As demonstrated in the research conducted by Chinese and European research centres [97,98], AR has the potential to play a pivotal role in the domain of employee training. By facilitating the creation of realistic simulations of hazardous situations within a controlled and secure environment, AR can contribute to the enhancement of training efficacy. In the field of mining, the integration of augmented reality technology facilitates the generation of three-dimensional visualisations of subterranean structures and corridor networks. This development has significant implications for the realm of mining operation planning, offering enhanced efficiency and responsiveness in crisis situations. Furthermore, the utilisation of augmented reality enables operators to undertake remote analysis of data collected by sensors deployed within the mine environment, thereby mitigating the necessity for physical presence in hazardous zones [99].

In the construction industry, AR is used for a variety of purposes, including the overlaying of technical plans on real structures. This process enables the detection of potential execution errors before they physically arise. The technology has been demonstrated to facilitate the identification of hazardous locations in real time during construction site operations. Such locations may include areas with an elevated risk of falls from height or the presence of hazardous equipment [100]. Furthermore, augmented reality solutions are being incorporated into safety management systems with increasing frequency, providing automatic visual and audible warnings to workers who are approaching hazardous areas. The integration of augmented reality with IoT and BIM systems has been demonstrated to facilitate the creation of comprehensive end-to-end environments. These environments have been shown to support occupational safety and operational efficiency. With regard to future projections, the AR market in the industrial sector—encompassing construction and mining—is anticipated to experience substantial growth. According to reports, the value of the augmented reality market for industrial applications is expected to exceed $30 billion by 2030. This growth will be driven not only by the development of hardware, such as next-generation AR glasses, but also by an increasing awareness of the operational and safety benefits of technology.

The integration of VR/AR systems with Internet of Things (IoT) and Big Data technologies represents a promising direction for future research. IoT-based wearable and environmental sensors can provide continuous streams of information regarding worker health, equipment status, and workplace hazards, while Big Data analytics enable the identification of hidden patterns and predictive modelling of risks. Embedding these data-driven insights into immersive VR/AR environments would allow the creation of dynamic training scenarios and real-time safety visualisations, thus supporting both proactive hazard prevention and adaptive decision-making on site. Such integration also contributes to the development of digital twins of mining and construction processes, providing a powerful tool for enhancing situational awareness and operational safety [101]. Blockchain and 6G technologies were not included in this review, as no studies have yet confirmed their practical implementation in mining or construction. Nevertheless, both areas represent promising directions for future research, as their potential applications may significantly contribute to enhancing efficiency, security, and safety management in these industries [102].

At the same time, the use of immersive and wearable technologies raises important ethical and legal concerns. VR/AR systems often collect sensitive biometric and behavioural data, which requires strict protection under frameworks such as GDPR to prevent misuse and surveillance of workers. Furthermore, issues of liability in case of system malfunction remain unresolved, highlighting the need for clear legal guidelines on the responsibilities of employers, developers, and manufacturers. The absence of specific certification standards for head-mounted displays and wearable exoskeletons further complicates their safe adoption. Addressing these ethical and legal aspects through regulatory frameworks, transparent communication, and participatory design is crucial to ensuring not only technical efficiency but also trust and fairness in the deployment of innovative safety technologies [103,104,105].

In view of the increasing significance of VR and AR, there is a necessity for further research to enhance occupational safety. It is recommended that future research directions concentrate on the following areas:

- Developing more advanced simulation models that take into account a greater number of environmental variables and dynamic human–machine interactions;
- Personalization of training with the use of artificial intelligence, which can adapt training scenarios to the individual needs and level of competence of users;
- Integration of VR/AR systems with Internet of Things and Big Data technologies, which will enable the creation of intelligent systems for warning and predicting threats in real time;
- Assessment of the long-term impact of the use of VR and AR on work efficiency and psychophysical health of employees;
- Analysis of ethical and legal aspects related to the use of virtual and augmented reality technology, including data privacy issues, liability for system errors, as well as technical and quality standards.

#### 4.1.2. Innovative Personal Protective Equipment and Collective Protective Equipment

Personal and collective protective equipment plays a fundamental and key role in ensuring occupational safety in any industry. Despite the prevalence of simple solutions to these problems, there is an increasing demand for more innovative solutions, which are often tailored to the specific needs of the industry in which they are employed (e.g., cooling vests for underground miners or helmets equipped with fall sensors for construction workers). Technological advances, the evolution of materials science and the integration of digital systems with traditional engineering solutions have facilitated the development of more advanced, effective and ergonomic forms of protection for workers exposed to various occupational hazards.

The implementation of innovative personal and collective protective measures is primarily aimed at increasing the effectiveness of protecting the health and life of employees. The advent of contemporary safety helmets, intelligent cooling systems, vital signs monitoring vests and toxic gas detection systems that are based on real-time data analysis has resulted in a marked enhancement in the level of safety, thereby enabling a swift response in emergency situations. Innovative PPE is distinguished by its enhanced user-friendliness, which in turn fosters greater acceptance among employees. The introduction of breathable, lightweight and resistant materials has resulted in a significant reduction in the burden of protective equipment during routine work activities [106]. This, in turn, has the effect of reducing the number of cases of improper use or complete omission of PPE. Furthermore, contemporary protective measures facilitate their integration with information technologies, thereby enabling continuous monitoring of working conditions and immediate alerting about danger. The continuous development of technologies, ranging from enhanced energy absorbers to intelligent personal protective equipment monitoring and detection systems, has engendered novel opportunities for the prevention of accidents and the enhancement of workers’ health protection [107].

The effective implementation of these measures requires a comprehensive analysis of risks, with consideration of employee perceptions, the elimination of organisational and financial barriers, and the consideration of individual user needs. Furthermore, contemporary protective equipment, which is equipped with electronic systems, has the potential to be unreliable in harsh environmental conditions (e.g., dust, humidity, etc.). Improper operation or misinterpretation of data can engender a false sense of security and result in a delayed response to a genuine threat. The implementation of advanced technologies necessitates the provision of adequate training for personnel and the integration of novel safety procedures, which can incur supplementary expenses and may encounter resistance from staff members, particularly in facilities exhibiting a low degree of digitisation.

Further research directions should focus on

- Developing environmentally resilient protective systems that will be able to operate reliably in extreme conditions of the working environment;
- The development of universal systems for the integration of data from various sources (sensors, cameras, biological data) in order to create coherent safety management systems;
- Research on the psychological and social aspects of the acceptance of new collective and individual protection measures by employees and the development of effective training and implementation strategies.

#### 4.1.3. Exoskeletons

Exoskeletons represent a novel solution for enhancing the physical performance of employees in the construction and mining industries during their professional duties. The utilisation of these devices has been demonstrated to exert a substantial contribution to the enhancement of occupational safety and the mitigation of strain on the musculoskeletal system [108,109]. In the context of construction and mining, where high-risk work is performed, often in a forced body position and with high physical loads, exoskeletons are a potential tool to reduce the risk of injuries and accidents. The primary benefits of exoskeletons are the reduction in muscle fatigue and pain resulting from overload. The distribution of forces and support for body movements is achieved by these devices, thereby reducing strain on the spine, joints and muscles, and consequently minimising the risk of overload injuries [85,108,109]. Furthermore, the enhancement of work ergonomics through the integration of exoskeletons has been demonstrated to engender an augmentation in efficiency, whilst concomitantly extending the duration of specific activities without compromising the physical condition of the employee.

The use of exoskeletons is associated with certain risks and limitations. A number of studies have been conducted on the use of exoskeletons in recent years; however, many of them are characterised by a limited sample size, ranging from just four participants [82] to a maximum of forty-one [91]. Such small study groups may not provide sufficient representativeness of the results, making it difficult to draw general conclusions on a wider population of construction workers. In addition, most of these studies were carried out under controlled laboratory conditions, which do not always reflect the complexity and dynamics of real construction sites or mines.

The current literature highlights the necessity for precise design and continuous improvement of exoskeleton construction to minimise potential undesirable side effects. For instance, a reduction in the load on one muscle group may lead to an increase in the load on other groups, as noted by [79,80,81]. Furthermore, concerns have been raised regarding the compatibility of exoskeletons with existing safety measures on construction sites, including protective harnesses, which may limit their practical application [80,81]. A further significant restriction pertains to the paucity of research that considers the long-term implications of exoskeletons and their effect on the well-being and health of users. The majority of extant research has focused on short-term experiments, without analysing the potential effects of many months or years of use of these devices. This is a crucial aspect for evaluating the practical usefulness of exoskeletons in everyday work. It is evident that further research is required in order to ascertain the long-term impact of exoskeleton use on workers’ health, as well as to determine the extent of user acceptance of this technology.

In addition, it is imperative to investigate the potential incorporation of exoskeletons into prevailing occupational health and safety processes and procedures. In order to enhance comprehension of the practical aspects inherent in the implementation of this technology, it is imperative that real-world field trials are conducted. It is recommended that future research efforts concentrate on the adaptation or development of novel exoskeleton solutions that are tailored to the particular requirements of specific industries, such as construction and mining. These solutions have the potential to enhance safety and productivity among workers. In addition, exoskeletons must be designed to facilitate integration with other elements of the employee’s equipment. Furthermore, it is essential to ensure that they are adapted to work in confined spaces, such as narrow shafts and mine environments. The devices must be adjustable in terms of fit and level of support, durable, easy to clean, and must not hinder workers in their fundamental tasks.

Research also indicates the considerable impact of exoskeletons on the cognitive functions of workers, which poses a significant challenge for designers and manufacturers of these devices. In the context of forthcoming research and development initiatives, a primary objective should be to mitigate this effect. This is imperative to ensure that the integration of exoskeletons into routine practice will yield the anticipated benefits without compromising the health and well-being of users.

In the context of future research on the use of exoskeletons, several significant directions can be indicated:

- Research on the long-term impact of their use on the health of employees, including the analysis of biomechanical and adaptive changes in the body;
- The development of technologies enabling individual adjustment of exoskeletons to the body structure of the user and the specifics of the tasks performed;
- The integration of exoskeletons with health and work environment monitoring systems, which will enable the creation of a comprehensive employee protection system.

### 4.2. Inter-Technological Integration—Opportunities and Limitations

Recent technological advancements have provided a range of opportunities to enhance occupational safety and health conditions, particularly in high-risk sectors such as mining and construction. The advent of innovative solutions, encompassing virtual and augmented reality, unmanned aerial vehicles and inspection robots, the Internet of Things and intelligent sensor systems, modern personal and collective protective equipment, exoskeletons and artificial intelligence, has precipitated a paradigm shift in the realm of occupational safety management. The utilisation of these technologies, whether in isolation or in conjunction with one another, facilitates the precise monitoring of hazards and the analysis of working conditions and the working environment. In addition, these technologies enable the prediction and effective prevention of dangerous situations. The potential of combining these technologies, as well as the limitations of such combinations, are summarised in Table 2.

#### 4.2.1. Opportunities on the Use and Mixing of Modern Technologies

The integration of innovative technologies to enhance occupational safety in the mining and construction sectors presents numerous opportunities. The integration of virtual reality and augmented reality with exoskeletons represents a promising, albeit nascent, synergy in the context of enhancing occupational safety in high-risk sectors. The integration of VR and AR with exoskeletons facilitates the acquisition of optimal techniques and attitudes for professional tasks, enhancing safety and ergonomics in the workplace. For instance, the seamless integration of reality and virtual training has been demonstrated to enhance the efficiency of equipment utilisation and mitigate the risk of injuries arising from incorrect posture or excessive exertion. The utilisation of augmented reality has been demonstrated to facilitate the visualisation of optimal working postures, ranges of motion and load limits for the operator of the exoskeleton. Augmented reality has the capacity to display visual alerts in the employee’s field of vision when their posture exceeds safe parameters. Furthermore, the utilisation of VR and AR simulations facilitates the collection of data pertaining to employee reactions during the implementation process. Simulations enhanced by exoskeletons promise to yield novel insights into physical loads, thereby facilitating the optimisation of technological processes.

VR and AR systems have the capacity to monitor the technical condition and correct use of PPE and CPE. For instance, sensors integrated into AR helmets or glasses can monitor the integrity of the helmet, the level of wear of protective gloves, or the correct fastening of safety harnesses. The transmission of this data to a control centre in real time facilitates the expeditious implementation of remedial measures in the event of any irregularities. In the context of CPE, AR can display information on the technical condition of scaffolding, excavation safety measures or ventilation systems, facilitating ongoing inspection and maintenance. The integration of VR and AR has the potential to facilitate the development of more ergonomic and functional PPE. Smart helmets, equipped with integrated augmented reality displays, serve as a prime example of this technological integration. These helmets eliminate the necessity for workers to carry additional devices, such as tablets, thereby reducing their physical and psychological workload. In the future, there is the potential for the integration of equipment with active properties and sensors with VR and AR technology, thereby enabling the dynamic adjustment of protection levels to changing conditions. Furthermore, the integration of VR and AR with PPE and CPE holds considerable potential in the domain of simulation testing of protective equipment and digital assessment of the suitability of protective equipment through integrated training scenarios.

Exoskeletons that function in conjunction with PPE and CPE have the capacity to mitigate the risk of injuries related to overload, in addition to providing support and monitoring worker overload. The reduction in operator fatigue enables prolonged work without a decline in concentration and precision, which results in enhanced task performance. The integration of exoskeletons with protective equipment such as helmets, goggles, ear muffs, gloves and protective footwear is a potential avenue for enhancing ergonomic support and safety.

The use of unmanned aerial vehicles (UAVs) and inspection robots, due to their mobility and capacity for real-time data collection, facilitates the inspection and monitoring of inaccessible areas. In a similar manner, sensors have the capacity to collect data in real time, which is subsequently transmitted to the operator for analysis. The application of UAVs and inspection robots, in conjunction with data from sensors, facilitates the acquisition of data (e.g., images, scans, measurement data) for the creation of realistic 3D environments and accident reconstructions, which are then displayed in VR. The utilisation of a combination of augmented reality, virtual reality (VR) unmanned aerial vehicles and inspection robots or sensors enables operators to observe the environment from the perspective of a UAV or robot in real time [110]. To illustrate this point, during the inspection of a mine wall, a UAVs equipped with a camera transmits images to the operator’s virtual reality goggles. The operator, situated within a secure control centre, is able to meticulously examine the structure of the wall, thereby identifying cracks, landslides, and other potential hazards. In the field of construction, the employment of UAVs has been instrumental in facilitating the monitoring of progress in elevated areas or in locations that are otherwise challenging to access, such as bridges and chimneys. In addition, the integration of augmented reality with unmanned aerial vehicles and inspection robots or sensors enables remote control of devices, thereby increasing the efficiency of inspection work. Employees have the capacity to operate robots utilising augmented reality interfaces, thereby enabling them to execute tasks with precision in challenging or hazardous environments. The utilisation of unmanned aerial vehicles in conjunction with robotic systems and sensors facilitates the acquisition of data, which can then be instantaneously exhibited within a virtual workspace. This capability enables expedited decision-making processes in instances where issues emerge. The amalgamation of these technologies with artificial intelligence facilitates real-time analysis of the collected data. The utilisation of artificial intelligence in the identification of patterns and potential hazards has become a subject of increasing importance in the field of accident prevention. Furthermore, the integration of artificial intelligence (AI) with VR and AR facilitates the personalisation of VR and AR scenarios to user requirements, the analysis of VR and AR technology users’ behaviour and reactions, and the subsequent risk assessment based on these analyses.

The potential for increased safety in mining and construction through the integration of modern personal and collective protective equipment with UAVs, inspection robots, the Internet of Things (IoT), sensors and artificial intelligence is a promising development. The employment of contemporary personal and collective protective equipment, in conjunction with UAVs or inspection robots, will facilitate real-time risk monitoring and evaluation. Conversely, sensors facilitate real-time monitoring and analysis of the working environment. The IoT technology has the capacity to facilitate continuous monitoring of employees’ health. Sensors can be utilised to measure employees’ vital signs, including body temperature, oxygen saturation, and other physiological indicators [111]. The results of these measurements can be tracked in real time and used to respond quickly to threats. This will serve to significantly reduce the risk of serious incidents in the workplace. The integration of innovative protective measures with AI systems and sensors has the potential to automate work condition monitoring processes. The integration of the Internet of Things with sensors, in conjunction with artificial intelligence, facilitates continuous monitoring of the working environment. In the event of dangerous conditions being detected, these systems have the capacity to automatically notify employees of the hazard and control evacuation procedures.

The integration of the Internet of Things (IoT) and sensors with exoskeletons is of significant importance in ensuring ergonomic working conditions. These technologies facilitate the analysis of biomechanical data, health and fatigue monitoring during the use of exoskeletons, while providing physical and ergonomic support. Conversely, the integration of AI with exoskeletons facilitates enhanced functionality, promoting ergonomic interaction with the user and optimising movement mechanics. For instance, exoskeletons can be integrated with AI systems that continuously monitor the user’s posture and load, adjusting their range of motion to the individual needs and limitations of the worker. Such solutions have been shown to have the potential to significantly reduce the risk of strain injuries.

Recent studies confirm the effectiveness of combining different innovative technologies to improve occupational safety. For example, the integration of augmented reality with BIM and IoT enables real-time visualisation of sensor data directly on construction sites, which significantly improves situational awareness and supports faster decision-making regarding occupational safety [112]. Similarly, artificial intelligence has been successfully integrated with personal protective equipment monitoring systems, where computer vision algorithms automatically detect the correct use of helmets, vests, or safety glasses, reducing the need for manual inspections and increasing compliance with safety procedures [113,114]. These examples illustrate that inter-technological integration can deliver tangible benefits in terms of efficiency, safety awareness and risk reduction in both mining and construction. Whereas recent studies in the mining sector confirm the benefits of integrating multiple innovative technologies to enhance safety and operational efficiency. For instance, Zhang [115] and Dodoo et al. [116] demonstrated that a safety monitoring system combining BIM, IoT and artificial intelligence significantly improved real-time hazard detection, decision support, and emergency response in smart mining environments. Moreover, Singh et al. [117] and Rodriguez et al. [118] demonstrated the integration of UAVs equipped with LiDAR and VR/AR technologies, enabling precise surface mapping and operations planning in open-pit mining — thereby improving situational awareness and operational safety. These examples illustrate the tangible advantages of technological integration in enhancing mining safety and facilitating proactive risk management.

#### 4.2.2. Limitations on the Use and Mixing of Modern Technologies

The application and integration of technological solutions are subject to inherent limitations. A primary challenge confronting the industry is the substantial financial outlay required for investment, in addition to the ongoing costs of maintenance and servicing. A significant proportion of the technologies utilised necessitate periodic servicing and calibration, a factor that has been demonstrated to exert a substantial influence on the cost-effectiveness of technology implementation. Moreover, in numerous instances, the cost-effectiveness of investing in contemporary technologies is constrained by protracted return on investment cycles and an absence of comprehensive comprehension regarding the advantages of such solutions. Therefore, a financial assessment of the implementation becomes important.

Employee opposition to the implementation and utilisation of contemporary technologies has the potential to engender resistance from employees who harbour concerns regarding the discomfort associated with the wearing of equipment or the lack of confidence in novel solutions. However, the reluctance of employees to embrace technology, coupled with concerns regarding privacy, has the potential to impede the integration of contemporary systems [119]. The successful implementation of modern technologies frequently necessitates alterations in the organisational culture of a company. It is imperative to establish an environment that is receptive to innovation and fosters continuous enhancement and learning. A paucity of management commitment, inadequate communication with employees, or insufficient integration of new technologies into existing work processes can result in the marginalisation or inefficient use of these technologies. The transition from a reactive to a proactive approach to safety is a pivotal factor in achieving successful technology implementation.

Another significant limitation to the use of equipment (modern protective measures, exoskeletons) may be the size and weight of these technological solutions. The equipment can be heavy and bulky, which can restrict the employee’s freedom of movement, cause discomfort and even fatigue, paradoxically increasing the risk of accidents. Moreover, the aggregation of data concerning employee movements, activities and performance through the utilisation of contemporary technologies gives rise to questions and concerns regarding privacy and the potential utilisation of this data for excessive control. It is also important to note that the collection and analysis of user data, including intensive monitoring of employees, may result in legal and ethical issues. It is imperative to acknowledge these issues, as failure to do so may result in the erosion of employee trust and the exposure of companies to potential legal challenges. Consequently, when implementing innovative technologies (e.g., physiological monitoring technologies), employers must take ethical and privacy issues into account. It is imperative that these aspects are regulated in accordance with the prevailing legislation, whilst ensuring the protection of the interests of both parties. Additionally, systems that collect information from multiple sources are vulnerable to cyber attacks, which can have serious consequences. It is therefore essential to develop effective strategies to protect these systems from potential threats.

Despite their dynamic development, many technologies are still evolving and may have technical limitations in specific mining or construction conditions. Therefore, another significant aspect impacting the usability of contemporary technologies is their resistance to the environmental conditions in which they are employed. Electronic equipment is particularly vulnerable to critical conditions (moisture, dust, vibrations, extreme temperatures) that are prevalent in the mining and construction industries. In the field of construction, the most significant challenges are often associated with inclement weather conditions, such as strong winds and variable temperatures. In the field of mining, however, environmental factors differ significantly from those encountered in construction. Consequently, technological adaptation must be meticulously tailored to these distinct factors and industry requirements. In the context of mining operations, the most significant challenges encountered by professionals include elevated temperatures, humidity, dust, vibrations, natural shocks, and inadequate communication. It is imperative to acknowledge that the environmental conditions associated with underground and opencast mining will diverge. The selection of technology must be made with consideration for the environmental conditions in which it will be employed.

The necessity to equip personnel with the requisite competencies to utilise contemporary tools represents a further constraint on the integration of modern technologies. Research has demonstrated that a paucity of training can engender feelings of anxiety and uncertainty, which can have a detrimental effect on the utilisation of technology [120]. Technologies should be designed with ease of use in mind to minimise stress associated with new tools and increase employee engagement [121].

Despite the significant potential of inter-technological integration, recent studies highlight serious barriers and limitations to combining innovative solutions in construction and mining. Research shows that exoskeletons may interfere with fall protection harnesses and other PPE, making their joint use problematic in practice [122]. Moreover, while exoskeletons can reduce physical strain, they often increase cognitive workload by up to 33%, which can offset ergonomic benefits and raise the risk of errors [74]. Similar compatibility challenges have been reported in the integration of AR/VR with safety requirements: field tests of HoloLens indicated that the device did not meet safety standards for protective eyewear, while misalignment artefacts reduced the reliability of quality control tasks [21]. Other studies underline the discomfort and cybersickness associated with intensive VR training in mining, limiting its applicability in extended safety programmes [22,23]. Finally, the simultaneous use of multiple PPE elements, such as helmets, respirators, and protective glasses, has been shown to reduce visibility and comfort, which complicates integration with AR headsets or wearable monitoring systems [123]. These findings indicate that although integration of emerging technologies is promising, their practical application requires careful testing, adaptation to harsh working environments, and the development of standards to ensure both safety and usability.

#### 4.2.3. Practical Ways to Reduce the Risks on the Use and Mixing of Modern Technologies

Although the integration of augmented and virtual reality (AR/VR), personal and collective protective equipment (PPE/CPE), and exoskeletons offers clear benefits for safety in mining and construction, their combined application also brings several risks, which can be mitigated through practical measures. First, ensuring compatibility with existing PPE standards is essential, as studies have reported frequent conflicts between exoskeleton straps or AR headsets and conventional protective equipment, especially fall-arrest harnesses and helmets [21,122]. Manufacturers should therefore adopt integrated design approaches and test new systems for interoperability before large-scale deployment. Second, managing cognitive and physical load is crucial, since exoskeletons and AR/VR may reduce biomechanical strain but simultaneously increase mental effort, thereby affecting situational awareness [124,125]. Adaptive systems that filter information and adjust exoskeleton support to the worker’s state can help balance these effects. Third, discomfort and user acceptance remain major obstacles, as prolonged VR exposure may cause cybersickness and resistance to new technologies in training or operations; gradual implementation and participatory design, where end-users contribute to system adjustments, are effective ways to improve usability [22,23]. Fourth, ethical and legal safeguards are required because AR/VR and wearable technologies collect sensitive data, making compliance with GDPR and clear regulations on liability in case of malfunctions necessary [123]. Finally, continuous evaluation and feedback loops through pilot programmes, safety audits, and structured reporting from workers are key to iteratively improving systems and preventing unintended risks [15]. Together, these measures demonstrate that while integration of modern technologies is challenging, their risks can be substantially reduced through proactive technical, organisational, and legal interventions supported by continuous worker engagement.

## 5. Conclusions

Modern technological developments have been found to have a significant impact on improving occupational safety in sectors characterised by a high level of occupational risk, such as mining and construction. Employees in these industries are particularly vulnerable to the occurrence of occupational accidents and the long-term impact of harmful environmental factors. Consequently, the implementation of innovative technological solutions is becoming a pivotal component of preventive strategies to protect the health and lives of workers.

Innovative technologies in mining and construction have been identified as key elements in the development of more effective occupational safety management strategies. The integration of automation, digitisation, data analysis and intelligent monitoring systems establishes a cohesive system that responds to risks and actively prevents them. In the face of mounting legal, social and economic pressures, investment in technological innovation has emerged not only as a manifestation of concern for workers, but also as a prerequisite for the sustainable development of these key industries.

In consideration of the analyses that have been conducted, it can be concluded that modern technologies, including but not limited to virtual and augmented reality, unmanned aerial vehicles, inspection robots, the Internet of Things, artificial intelligence, and exoskeletons, offer significant potential for improving occupational safety in high-risk industries, particularly in the construction and mining sectors. These technologies facilitate not only realistic training and precise inspections, but also real-time monitoring of the work environment and physical support for employees. Nevertheless, significant challenges persist, encompassing concerns regarding reliability, interoperability, cybersecurity, and employee acceptance and training. The integration of these technologies into cohesive safety management systems has the potential to result in a substantial reduction in workplace accidents.

Further research should include the development of existing technologies and the exploration of new and more effective solutions. Effective implementation also requires further interdisciplinary research, including social, legal, and ethical aspects, to fully harness the opportunities offered by the digital transformation of work environments.

This two-part study has shown that innovative technologies in mining and construction should be understood not only as novel solutions, but as tools that bring measurable safety, social, and economic benefits through effective implementation. By jointly analysing these two high-risk sectors, it has been demonstrated that, despite their specific conditions, both industries face comparable challenges and can benefit from transferable practices and synergies. Positive examples of technological integration, such as AR with BIM and IoT or AI-supported PPE monitoring, confirm tangible improvements in situational awareness and compliance, while identified limitations—including compatibility issues, cognitive load, cybersickness, and ethical dilemmas—highlight the need for careful adaptation. Practical recommendations such as modular PPE design, adaptive information filtering, and structured user feedback may reduce these risks, paving the way for broader adoption. Finally, future research should focus on the convergence of VR/AR with IoT and Big Data, as well as on emerging areas such as blockchain and 6G, accompanied by the development of robust ethical and legal frameworks to safeguard workers’ rights and data privacy.

## Figures and Tables

**Table 1:** Keywords and search results (own study).

Technology	Keywords			
	Mining Engineering	Results	Civil Engineering	Results
Augmented reality (AR) and virtual reality (VR)	VR OR virtual reality OR AR OR augmented reality AND mining engineering OR mining AND occupational safety	31	VR OR virtual reality OR AR OR augmented reality AND civil engineering OR construction industry AND occupational safety	29
Innovative personal protective equipment (PPE) and collective protective equipment (CPE)	new OR modern OR innovative AND personal protective equipment OR individual protective equipment OR collective protective equipment OR group protective equipment AND mining OR mining engineering	22	new OR modern OR innovative AND personal protective equipment OR individual protective equipment OR collective protective equipment OR group protective equipment AND civil engineering OR construction industry	41
Exoskeletons	exoskeleton AND mining OR mining engineering	2	exoskeleton AND construction industry OR civil engineering	29

**Table 2:** Analysis of the possibilities of combining modern technologies in mining and construction—opportunities and limitations (own study).

	Unmanned Aerial Vehicles and Inspection Robots	IoT and Sensors	Artificial Intelligence	Augmented Reality (AR) and Virtual Reality (VR)	Innovative Personal Protective Equipment (PPE) and Collective Protective Equipment (CPE)	Exoskeletons
	Opportunities					
Exoskeletons	Management of hazardous and emergency situations	Biomechanical data analysis; Health and fatigue monitoring; Physical and ergonomic support	Assist with high-risk physical work; Motor support; Managing fatigue and interruptions	Haptic feedback; Synchronisation of motion data	Motion support and increased worker safety	
Innovative personal protective equipment (PPE) and collective protective equipment (CPE)	Real-time monitoring and risk assessment	Monitor and analyse the work environment in real time; Manage the operation and condition of protective equipment; Responding to hazardous and accident situations; Data analysis and prediction of hazards and accidents	Creating smart PPE and CPE; Automation of collective protection system	Compatibility of purpose with actual activities performed; Test simulations of PPE and CPE.		Lack of standards and norms; Compatibility problems with security systems
Augmented reality (AR) and virtual reality (VR)	Creating realistic 3D environments; Reconstructing accidents; Monitoring working conditions and identifying hazards; Use of real-time AR in work environments	Integrating data from IoT devices into a VR environment; Industrial training and simulation	Machine learning for VR environment adaptation; Personalization of VR/AR scenarios; Analysis of user response behaviour of VR/AR technologies; Risk assessment		A false sense of security; Ergonomic and health problems	Cybersecurity Threats; Technical errors and physical consequences
Artificial intelligence	Monitoring working conditions and identifying hazards; Predicting accidents and failures; Mapping and 3D modelling of the work environment	Real-time monitoring and analysis of the work environment; Predicting accidents and failures; Access control and employee identification		Algorithmic errors; Improper data analysis; Data leakage; Invasion of privacy; Qualified personnel requirement	Algorithmic errors; Improper data analysis; Data leakage; Invasion of privacy; Cybersecurity threat	Algorithmic errors and improper data analysis; Data leakage; Invasion of privacy; Cybersecurity threat; The complexity of technology integration
IoT and sensors	Real-time monitoring of the work environment; Remote inspection and control of hazardous sites; Data analysis and prediction of hazards and accidents		Reducing employee vigilance; AI misinterpretation of data; Data leakage; Invasion of privacy; Cybersecurity threat; Ethical and legal issues	Data leakage; Invasion of privacy; Cybersecurity threat	Unreliability of technology; Data leakage; Invasion of privacy; Cybersecurity threat; Need for qualified personnel	A false sense of security; Unreliability of technology; Cybersecurity threat; The complexity of technology integration
Unmanned aerial vehicles and inspection robots		Data leakage; Invasion of privacy; Cybersecurity threat; The complexity of technology integration; Unreliability of technology; Dependencies on communication systems	Algorithmic errors; Improper data analysis; Data leakage; Invasion of privacy; Cybersecurity threat; The complexity of technology integration	A false sense of security; Technical issues and hardware compatibility; Cybersecurity threat; Data leakage; Invasion of privacy; Need for qualified personnel	A false sense of security; Compatibility issues; Cybersecurity threat	Lack of standards and norms; Threats to cybersecurity; Complexity of technology integration
	Limitations					

## Data Availability

The original contributions presented in this study are included in the article. Further inquiries can be directed to the corresponding author.

## Abbreviations

The following abbreviations are used in this manuscript:

OSH	Occupational Safety and Health
AR	Augmented Reality
VR	Virtual Reality
PPE	Personal Protective Equipment
CPE	Collective Protective Equipment
EMS	Emergency Medical Services
IoT	Internet of Things
BIM	Building Information Modelling
WRMSDs	work-related musculoskeletal disorders

## References

[1] Al-Bayati A.J. (2021). Impact of Construction Safety Culture and Construction Safety Climate on Safety Behavior and Safety Motivation. Safety.

[2] Sidani A., Poças Martins J., Soeiro A. (2023). Catalysing Construction Safety: A Comparative Analysis of Technological Advancements across High-Risk Industries. Buildings.

[3] Haleem A., Javaid M., Singh R.P. (2025). Encouraging Safety 4.0 to enhance industrial culture: An extensive study of its technologies, roles, and challenges. Green Technol. Sustain..

[4] Arana-Landín G., Laskurain-Iturbe I., Iturrate M., Landeta-Manzano B. (2023). Assessing the influence of industry 4.0 technologies on occupational health and safety. Heliyon.

[5] Berglund L., Johansson J., Johansson M., Nygren M., Stenberg M. (2023). Exploring safety culture research in the construction industry. WORK.

[6] Guzman J., Recoco G.A., Pandi A.W., Padrones J.M., Ignacio J.J. (2022). Evaluating workplace safety in the oil and gas industry during the COVID-19 pandemic using occupational health and safety Vulnerability Measure and partial least square Structural Equation Modelling. Clean. Eng. Technol..

[7] Bęś P., Strzałkowski P. (2024). Analysis of the Effectiveness of Safety Training Methods. Sustainability.

[8] Protasenko O., Mygal G., Kobrina N., Mykhailova E. (2023). Ergonomic thinking in the design of human-machine systems. Bull. Natl. Tech. Univ. «KhPI» Ser. New Solut. Mod. Technol..

[9] Moore N. (2006). Desk research. How to Do Research: The Practical Guide to Designing and Managing Research Projects.

[10] Topolewski S., Górnikiewicz M., Stawarz P. (2023). The literature review and the “desk research” methods in studies conducted in social sciences with particular emphasis on security, political, and international relations studies. Stud. Wschod..

[11] Bednarowska Z. (2013). Desk research—Wykorzystanie potencjału danych zastanych w prowadzeniu badań marketingowych i społecznych. Zesz. Prasozn..

[12] Gupta R.K. (2024). Methodological and Theoretical Rigor in Desk Research. ResearchGate. https://www.researchgate.net.

[13] Azhar S. (2017). Role of visualization technologies in safety planning and management at construction jobsites. Procedia Eng..

[14] Yap J.B.H., Lee K.P.H., Wang C. (2023). Safety enablers using emerging technologies in construction projects: Empirical study in Malaysia. J. Eng. Des. Technol..

[15] Strzałkowski P., Bęś P., Szóstak M., Napiórkowski M. (2024). Application of Virtual Reality (VR) Technology in Mining and Civil Engineering. Sustainability.

[16] Eiris R., Gheisari M., Esmaeili B. (2018). PARS: Using augmented 360-degree panoramas of reality for construction safety training. Int. J. Environ. Res. Public Health.

[17] Lukhele B.N., Laseinde O.T. (2024). Exploring Mechanical Engineering Equipment at TVET Colleges in South Africa, Towards Integrating Virtual and Cyber-Physical Learning. Procedia Comput. Sci..

[18] Bellanca J.L., Orr T.J., Helfrich W.J., Macdonald B., Navoyski J., Demich B. (2019). Developing a Virtual Reality Environment for Mining Research. Min. Metall. Explor..

[19] Assfalg J., Del Bimbo A., Vicario E. (2002). Using 3D and Ancillary Media to Train Construction Workers. IEEE Multimed..

[20] Stothard P., Laurence D. (2014). Application of a large-screen immersive visualisation system to demonstrate sustainable mining practices principles. Min. Technol..

[21] Kim H., Choi Y. (2019). Performance Comparison of User Interface Devices for Controlling Mining Software in Virtual Reality Environments. Appl. Sci..

[22] Pedram S., Palmisano S., Miellet S., Farrelly M., Perez P. (2022). Influence of age and industry experience on learning experiences and outcomes in virtual reality mines rescue training. Front. Virtual Real..

[23] Mitra R., Saydam S. (2013). Using Virtual Reality In Tertiary Education. Int. J. Technol. Learn..

[24] Chen Z., Liu L., Qi X., Geng J. (2016). Digital Mining Technology-Based Teaching Mode for Mining Engineering. Int. J. Emerg. Technol. Learn..

[25] Zhang H. (2017). Head-mounted display-based intuitive virtual reality training system for the mining industry. Int. J. Min. Sci. Technol..

[26] Dorey F., Knights P.F. (2015). Quantifying the benefits of simulator training for dragline operators. Min. Technol..

[27] Liao J., Lu G. (2009). Research on key techniques of virtual reality applied in mining industry. J. Coal Sci. Eng. China.

[28] McMahon M., Garrett M. (2010). Computer-Generated Three-Dimensional Training Environments: The Simulation, User, and Problem-Based Learning SUPL Approach. Int. J. Gaming Comput. Mediat. Simul..

[29] Tripathy D. (2014). Virtual reality and its applications in mining industry. J. Mines Met. Fuels.

[30] Onifade M., Said K.O., Shivute A.P. (2023). Safe mining operations through technological advancement. Process. Saf. Environ. Prot..

[31] Schaum A., Nieto A., Karmis M., Schafrik S.J. (2008). Haul Truck Safety and Virtual Environments Training. Min. Eng..

[32] Webber-Youngman R.C.W., van Wyk E.A. (2013). Incident Reconstruction Simulations-Potential Impact on the Prevention of Future Mine Incidents. J. S. Afr. Inst. Min. Metall..

[33] Nickel C., Knight C., Langille A., Godwin A. (2019). How Much Practice Is Required to Reduce Performance Variability in a Virtual Reality Mining Simulator?. Safety.

[34] Gürer S., Surer E., Erkayaŏglu M. (2023). Mining-Virtual: A Comprehensive Virtual Reality-Based Serious Game for Occupational Health and Safety Training in Underground Mines. Saf. Sci..

[35] Whyte J., Bouchlaghem N., Thorpe A., McCaffer R. (2000). From CAD to Virtual Reality: Modelling Approaches, Data Exchange and Interactive 3D Building Design Tools. Autom. Constr..

[36] Whyte J. (2003). Industrial Applications of Virtual Reality in Architecture and Construction. Electron. J. Inf. Technol. Constr..

[37] Dobrucali E., Demirkesen S., Sadikoglu E., Zhang C., Damci A. (2022). Investigating the impact of emerging technologies on construction safety performance. Eng. Constr. Archit. Manag..

[38] Acuña L.A., Rojas B.H., Reyes H.P., Arabia J.H., Piñero Pérez P.Y., Pupo I.P., Piñero Pérez P.Y., Kacprzyk J., Bello Pérez R., Pupo I.P. (2024). Systematic Review of Augmented Reality (AR) and Bim for the Management of Deadlines, Costs and Quality. Computational Intelligence in Engineering and Project Management.

[39] Eiris R., Jain A., Gheisari M., Wehle A. (2020). Safety immersive storytelling using narrated 360-degree panoramas: A fall hazard training within the electrical trade context. Saf. Sci..

[40] Rwamamara R., Norberg H., Olofsson T., Lagerqvist O. (2010). Using visualization technologies for design and planning of a healthy construction workplace. Constr. Innov..

[41] Le Q.T., Pedro A., Park C.S. (2015). A Social Virtual Reality Based Construction Safety Education System for Experiential Learning. J. Intell. Robot. Syst..

[42] Liang Z., Zhou K., Gao K. (2019). Development of Virtual Reality Serious Game for Underground Rock-Related Hazards Safety Training. IEEE Access.

[43] Hethmon T.A. (1997). Development of an Industrial Hygiene Program at a New Copper Mine in Chile. Appl. Occup. Environ. Hyg..

[44] Sakti M., Nirmalawati, Tawil S. (2022). Analysis of the causes of occupational accidents in nickel mining activities in Morowali. J. Eng. Appl. Sci..

[45] Qiao H. (2024). Research on the Application of Digital Twin Model Calculation and Updating Methods in Mining Engineering. Smart Innov. Syst. Technol..

[46] Garcia Gonzalez H., García-Ordiales E., Diez R.R. (2022). Analysis of the airborne mercury and particulate arsenic levels close to an abandoned waste dump and buildings of a mercury mine and the potential risk of atmospheric pollution. SN Appl. Sci..

[47] Adjiski V., Despodov Z., Mirakovski D., Serafimovski D. (2019). System architecture to bring smart personal protective equipment wearables and sensors to transform safety at work in the underground mining industry. Rud. Geol. Naft. Zb..

[48] Singh N., Gunjan K., Chaudhary G., Kaluri R., Victor N., Lakshmanna K. (2022). IoT enabled HELMET to safeguard the health of mine workers. Comput. Commun..

[49] Nipun D., Nath A., Behzadan H., Paal S.G. (2020). Deep learning for site safety: Real-time detection of personal protective equipment. Autom. Constr..

[50] Mahadi M.K., Rahad R., Mobassir M.S., Rahman A., Shafiullah A., Nishat M.M. Implementation of Personal Safety Equipment Tracking&Detection by DeepSORT&YOLOv8. Proceedings of the International Conference on Inventive Computation Technologies (ICICT).

[51] Calle R., Aguilar E., Hernández-García R., Barrientos R.J., Velastin S.A. (2024). An Uncertainty-Driven ScaledYOLOv4 for Open-Pit Mining Helmet Detection. Progress in Pattern Recognition, Image Analysis, Computer Vision, and Applications.

[52] Imam M., Baïna K., Tabii Y., Ressami E.M., Adlaoui Y., Boufousse S., Benzakour I., Abdelwahed E.H. (2025). Integrating real-time pose estimation and PPE detection with cutting-edge deep learning for enhanced safety and rescue operations in the mining industry. Neurocomputing.

[53] Günther F., Mischo H., Lösch R., Grehl S., Güth F., Mueller C. (2019). Increased safety in deep mining with IoT and autonomous robots. Mining Goes Digital.

[54] Nagarajapandian M., Ishana A., Jeevika K., Jefina S.J., Arun Mozhi Devan P. Health & Environment Monitoring and Alerting System for Mine Workers: From Proof of Concept to Experimental Validation. Proceedings of the 2023 IEEE International Conference on Sensors and Nanotechnology (SENNANO).

[55] Godwin A.A., Eger T.R. (2014). Ergonomic and usability ratings of helmets and head-mounted personal protective equipment in industry. Work.

[56] Szkudlarek J., Owczarek G., Jachowicz M., Zagrodny B., Sencerek J. (2023). Study of The Impact of Users’ Features on Dimensional Allowances Resulting from the Use of Personal Protective Equipment. Int. J. Environ. Res. Public Health.

[57] Huang X., Hinze J. (2003). Analysis of construction worker fall accidents. J. Constr. Eng. Manag..

[58] Spierings A.B., Stämpfli R. (2006). Methodology for the development of an energy absorber: Application to worker security ropes. Int. J. Impact Eng..

[59] Bogovski S., Lang I., Rjazanov V., Muzyka V., Tuulik V., Vitak A. (2007). Assessment of potential hazards during the process of house building in Estonia. Int. J. Environ. Health Res..

[60] Ulubeyli S., Kazaz A., Er B. (2014). Health and safety perception of workers in Turkey: A survey of construction sites. Int. J. Occup. Saf. Ergon..

[61] Anveden Berglind I., Lind M.L., Lidén C. (2012). Epoxy pipe relining—An emerging contact allergy risk for workers. Contact Dermat..

[62] Bello A., Xue Y., Gore R., Woskie S., Bello D. (2020). Exposures and urinary biomonitoring of aliphatic isocyanates in construction metal structure coating. Int. J. Hyg. Environ. Health.

[63] Teizer J.J. (2015). Wearable, wireless identification sensing platform: Self-Monitoring Alert and Reporting Technology for Hazard Avoidance and Training (SmartHat). Electron. J. Inf. Technol. Constr..

[64] Lo J.-H., Lin L.-K., Hung C.-C. (2023). Real-Time Personal Protective Equipment Compliance Detection Based on Deep Learning Algorithm. Sustainability.

[65] Bodtländer C., Helmus M., Kelm A., Meins-Becker A. BIM-Processes-Health & Safety. Proceedings of the International Structural Engineering and Construction.

[66] Borjan M., Patel T., Lefkowitz D., Campbell C., Lumia M. (2015). Assessing Barriers to the Use of Fall Protection in Small Residential Construction Companies in New Jersey. A J. Environ. Occup. Health Policy.

[67] Simpeh F., Bamfo-Agyei E., Amoah C. (2022). Barriers to the implementation of COVID-19 safety regulations: Insight from Ghanaian construction sites. J. Eng. Des. Technol..

[68] Kolegraff S., Parrotta K. (2023). Safety First Means Safety Fits: A Mixed Methods Analysis of Gender and Personal Protective Equipment Feminist Hackathon. New Trends Qual. Res..

[69] Okunola A., Afolabi A., Akanmu A., Jebelli H., Simikins S. (2024). Facilitators and barriers to the adoption of active back-support exoskeletons in the construction industry. J. Saf. Res..

[70] Okunola A., Akanmu A., Jebelli H. (2024). Fall risk assessment of active back-support exoskeleton-use for construction work using foot plantar pressure distribution. Adv. Eng. Inform..

[71] Al-Khiami M.I., Lindhard S.M., Wandahl S. (2024). Charting the Exoskeleton Industry: A Comprehensive Insight into Dynamics and Trends. IOP Conf. Ser. Earth Environ. Sci..

[72] Zeng Q., Tan G., Chen Q., Wu G., Xi T., An C. (2024). Design of topological configuration and key technologies for mining exoskeleton robots: Application and prospects of level set algorithm. Proc. SPIE—Int. Soc. Opt. Eng..

[73] Athar A., Vigilio F., Werner S., Sunil A.K. (2021). Systematic Review of Back-Support Exoskeletons and Soft Robotic Suits. Front. Bioeng. Biotechnol..

[74] Looze M.P., de Bosch T., Krause F., Stadler K.S., O’Sullivan L.W. (2015). Exoskeletons for Industrial Application and Their Potential Effects on Physical Work Load. Ergonomics.

[75] Alwasel A., Elrayes K., Abdel-Rahman E., Haas C. Reducing shoulder injuries among construction workers. Proceedings of the 29th International Symposium of Automation and Robotics in Construction.

[76] Golabchi A., Miller L., Rouhani H., Tavakoli M. Impact of Passive Back-Support Exoskeletons on Manual Material Handling Postures in Construction. Proceedings of the International Symposium on Automation and Robotics in Construction.

[77] Linner T., Pan M., Pan W., Taghavi M., Pan W., Bock T. Identification of usage scenarios for robotic exoskeletons in the context of the Hong Kong construction industry. Proceedings of the ISARC 2018—35th International Symposium on Automation and Robotics in Construction and International AEC/FM Hackathon: The Future of Building Things.

[78] Capitani S.L., Bianchi M., Secciani N., Pagliai M., Meli E., Ridolfi A. (2021). Model-based mechanical design of a passive lower-limb exoskeleton for assisting workers in shotcrete projection. Meccanica.

[79] Gonsalves N.J., Ogunseiju O.R., Akanmu A.A., Nnaji C.A. (2021). Assessment of a passive wearable robot for reducing low back disorders during rebar work. J. Inf. Technol. Constr..

[80] Gonsalves N.J., Khalid M., Akinniyi A., Akanmu A. Industry Perspectives of the Potential of Wearable Robot for Pipe Installation Work. Proceedings of the International Symposium on Automation and Robotics in Construction.

[81] Gonsalves N.J., Khalid M., Akinniyi A., Ogunseiju O., Akanmu A. Subjective Evaluation of Passive Back-Support Wearable Robot for Simulated Rebar Work. Proceedings of the International Symposium on Automation and Robotics in Construction.

[82] Bennett S.T., Han W., Mahmud D., Adamczyk P.G., Dai F., Wehner M., Veeramani D., Zhu Z. (2023). Usability and Biomechanical Testing of Passive Exoskeletons for Construction Workers: A Field-Based Pilot Study. Buildings.

[83] Ojha A., Gautam Y., Jebelli H., Akanmu A. (2024). Physiological impact of powered back-support exoskeletons in construction: Analyzing muscle fatigue, metabolic cost, ergonomic risks, and stability. Autom. Constr..

[84] Liu Y., Gautam Y., Ojha A., Shayesteh S., Jebelli H. (2024). Studying the Effects of Back-Support Exoskeletons on Workers’ Cognitive Load during Material Handling Tasks. Constr. Res. Congr..

[85] Breneman M., Ojha A., Jebelli H., Simkins S.J., Akanmu A. (2023). Breaking down barriers: A study of challenges to adopting powered exoskeletons in the US construction industry. Computing in Civil Engineering 2023: Data, Sensing, and Analytics—Selected Papers from the ASCE International Conference on Computing in Civil Engineering.

[86] Dunson-Todd M., Nik-Bakht M., Hammad A. Experimental Evaluation of Exoskeletons for Rebar Workers Using a Realistic Controlled Test. Proceedings of the International Symposium on Automation and Robotics in Construction.

[87] Baltrusch S.J., Krause F., de Vries A.W., de Looze M.P. (2024). Arm-support exoskeleton reduces shoulder muscle activity in ceiling construction. Ergonomics.

[88] Nnaji C., Ibrahim A., Okpala I. (2023). Semi-Active Exoskeletons for Forearm Muscle Strain Reduction. Computing in Civil Engineering 2023: Resilience, Safety, and Sustainability—Selected Papers from the ASCE International Conference on Computing in Civil Engineering.

[89] Mänttäri S., Rauttola A.P., Halonen J., Karkulehto J., Säynäjäkangas P., Oksa J. (2024). Effects of upper-limb exoskeleton on muscle activity in tasks requiring arm elevation: Part II—In-field experiments in construction industry settings. Work.

[90] Ibrahim A., Okpala I., Nnaji C., Akanmu A. (2024). Effects of using an active hand exoskeleton for drilling tasks: A pilot study. J. Saf. Res..

[91] Du B.B., Somasundram K.G., Johnston A., Bigelow P., Abdoli-Eramaki M., Jordan K.H., Yung M., Yazdani A. (2024). Skilled Workers’ Perspectives on Utilizing a Passive Shoulder Exoskeleton in Construction. Appl. Sci..

[92] Seo H., Pooladvand S., Aslanli A., Hasanzadeh S., Esmaeili B. (2023). Cognitive Impact of Wearing an Exoskeleton on Hazard Identification Performance of Construction Workers. Computing in Civil Engineering 2023: Resilience, Safety, and Sustainability—Selected Papers from the ASCE International Conference on Computing in Civil Engineering.

[93] Akanmu A., Okunola A., Jebelli H., Ammar A., Afolabi A. (2024). Cognitive load assessment of active back-support exoskeletons in construction: A case study on construction framing. Adv. Eng. Inform..

[94] Afolabi A., Yusuf A., Akanmu A. (2025). Predicting mental workload of using exoskeletons for construction work: A deep learning approach. J. Inf. Technol. Constr..

[95] Zheng L., Pan C., Wei L., Bahreinizad H., Chowdhury S., Ning X., Santos F. (2024). Shoulder-assist exoskeleton effects on balance and muscle activity during a block-laying task on a simulated mast climber. Int. J. Ind. Ergon..

[96] Rubenstone J. (2016). Mixing Reality on Site. ENR.

[97] Li X., Yi W., Chi H.-L., Wang X., Chan A.P.C. (2018). A critical review of virtual and augmented reality (VR/AR) applications in construction safety. Autom. Constr..

[98] Park C., Kim H., Cho Y.K. (2019). Framework of automated construction-safety monitoring using cloud-enabled BIM and BLE mobile tracking sensors. J. Constr. Eng. Manag..

[99] Xu W., Ma L., Xu C., Liu Y. (2021). Application of augmented reality to improve mine safety: A case study in a coal mine. Saf. Sci..

[100] Zhou Y., Whyte J., Sacks R. (2012). Construction safety and digital design: A review. Autom. Constr..

[101] Omrany H., Al-Obaidi K.M., Husain A., Ghaffarianhoseini A. (2023). Digital Twins in the Construction Industry: A Comprehensive Review of Current Implementations, Enabling Technologies, and Future Directions. Sustainability.

[102] Saah A.E.N., Yee J.-J., Choi J.-H. (2023). Securing Construction Workers’ Data Security and Privacy with Blockchain Technology. Appl. Sci..

[103] Giaretta A. (2025). Security and Privacy in Virtual Reality: A Literature Survey. Virtual Real..

[104] Hine E., Rezende I., Roberts H., Wong D., Taddeo M., Floridi L. (2024). Safety and Privacy in Immersive Extended Reality: An Analysis and Policy Recommendations. Digit. Soc..

[105] Roesner F., Kohno T., Molnar D. (2014). Security and Privacy for Augmented Reality Systems. Commun. ACM.

[106] Bhattacharjee S., Joshi R.K., Chughtai A.A., Macintyre C.R. (2019). Graphene Modified Multifunctional Personal Protective Clothing. Adv. Mater. Interfaces.

[107] Narayan Senapati J., Sharma V., Singh Choudhary A. (2022). Technological Advancements in Personal Protective Equipment: A Future Perspective. Health Leadersh. Qual. Life.

[108] Flor-Unda O., Casa B., Fuentes M., Solorzano S., Narvaez-Espinoza F., Acosta-Vargas P. (2023). Exoskeletons: Contribution to Occupational Health and Safety. Bioengineering.

[109] Botti L., Melloni R. (2024). Occupational Exoskeletons: Understanding the Impact on Workers and Suggesting Guidelines for Practitioners and Future Research Needs. Appl. Sci..

[110] Fross K., Szuliński T., Fross R. (2022). Mieszana rzeczywistość w projektowaniu BIM i inspekcjach budowli. BUILDER.

[111] Pawłowska Z. (2021). Szanse i zagrożenia dla bezpieczeństwa i higieny pracy związane z wdrażaniem w przedsiębiorstwach technologii Przemysłu 4.0. Occup. Safety. Sci. Pract..

[112] Getuli V., Capone P., Bruttini A., Isaac S. (2020). BIM-Based Immersive Virtual Reality for Construction Workspace Planning: A Safety-Oriented Approach. Autom. Constr..

[113] Fang W., Ding L., Luo H., Love P.E.D. (2018). Falls from Heights: A Computer Vision-Based Approach for Safety Harness Detection. Autom. Constr..

[114] Wu J., Cai N., Chen W., Wang H., Wang G. (2019). Automatic Detection of Hardhats Worn by Construction Personnel: A Deep Learning Approach and Benchmark Dataset. Autom. Constr..

[115] Zhang W. (2024). Research on the Application of Intelligent Technology in Mining Safety Monitoring. Highlights Sci. Eng. Technol..

[116] Dodoo J.E., Al-Samarraie H., Alzahrani A.I., Lonsdale M., Alalwan N. (2024). Digital Innovations for Occupational Safety: Empowering Workers in Hazardous Environments. Workplace Health Saf..

[117] Singh P., Murthy V.M.S.R., Kumar D., Raval S. (2024). A Comprehensive Review on Application of Drone, Virtual Reality and Augmented Reality with Their Application in Dragline Excavation Monitoring in Surface Mines. Geomat. Nat. Hazards Risk.

[118] Rodriguez J., Barakos G., Stothard P., Acosta Quelopana A.M. (2025). Mining Metaverse—Identifying Safety and Commercial Risks in Mining Operations. Mining.

[119] Codoceo-Contreras L., Rybak N., Hassall M. (2024). Exploring the impacts of automation in the mining industry: A systematic review using natural language processing. Min. Technol..

[120] Häikiö J., Kallio J., Mäkelä S., Keränen J.S. (2020). Iot-based safety monitoring from the perspective of construction site workers. Int. J. Occup. Environ. Saf..

[121] Yuan Y., Ye S., Lin L. (2021). Process monitoring with support of iot in prefabricated building construction. Sens. Mater..

[122] Howard J., Murashov V.V., Lowe B.D., Lu M.-L. (2020). Industrial Exoskeletons: Need for Intervention Effectiveness Research. Am. J. Ind. Med..

[123] Larson E.W. (2011). Barriers to the Use of Personal Protective Equipment. Occupational Health and Safety Management.

[124] Okunola A., Akanmu A., Jebelli H. (2024). Detection of Cognitive Loads during Exoskeleton Use for Construction Flooring Work. Proceedings of the Construction Research Congress.

[125] Govaerts R., Turcksin T., Vanderborght B., Roelands B., Meeusen R., De Pauw K., De Bock S. (2023). Evaluating Cognitive and Physical Work Performance: A Comparative Study of an Active and Passive Industrial Back-Support Exoskeleton. Wearable Technol..

